# *Candida auris* Updates: Outbreak Evaluation through Molecular Assays and Antifungal Stewardship—A Narrative Review

**DOI:** 10.3390/cimb46060362

**Published:** 2024-06-15

**Authors:** Silvia Ionescu, Ionut Luchian, Costin Damian, Ancuta Goriuc, Elena Porumb-Andrese, Cosmin Gabriel Popa, Roxana Gabriela Cobzaru, Carmen Ripa, Ramona Gabriela Ursu

**Affiliations:** 1 Department of Preventive Medicine and Interdisciplinarity (IX), Microbiology, Faculty of Medicine, “Grigore T. Popa” University of Medicine and Pharmacy, 700115 Iasi, Romaniaramona.ursu@umfiasi.ro (R.G.U.); 2 Department of Periodontology, Faculty of Dental Medicine, “Grigore T. Popa” University of Medicine and Pharmacy, 700115 Iasi, Romania; ionut.luchian@umfiasi.ro; 3 Department of Biochemistry, Faculty of Dental Medicine, “Grigore T. Popa” University of Medicine and Pharmacy, 700115 Iasi, Romania; 4 Department of Medical Specialties (III)—Discipline of Dermatology, Faculty of Medicine, “Grigore T. Popa” University of Medicine and Pharmacy, 700115 Iasi, Romania; 5 Department of Anatomy, Faculty of Medicine, “Grigore T. Popa” University of Medicine and Pharmacy, 700115 Iasi, Romania; 6 Microbiology Department, Gynecology and Obstetrics Hospital-Cuza Voda, 700038 Iasi, Romania

**Keywords:** *Candida auris*, host–fungal interaction, antifungal resistance, molecular diagnosis

## Abstract

*Candida auris* was reported by the WHO as second to *Cryptococcus neoformans*, in the list of nineteen fungal priority pathogens, along with two species with a new nomenclature, *Nakaseomyces glabrata* (*Candida glabrata*) and *Pichia kudriavzevii* (*Candida krusei*). This novel classification was based on antifungal resistance, the number of deaths, evidence-based treatment, access to diagnostics, annual incidence, and complications and sequelae. We assessed which molecular assays have been used to diagnose *Candida auris* outbreaks in the last five years. Using “*Candida auris*; outbreak; molecular detection” as keywords, our search in PubMed revealed 32 results, from which we selected 23 original papers published in 2019–2024. The analyzed studies revealed that the detection methods were very different: from the VITEK® 2 System to MALDI TOF (Matrix-Assisted Laser Desorption Ionization–Time of Flight), NGS (Next-Generation Sequencing), WGS (Whole Genome Sequencing), and commercially available real-time PCR (Polymerase Chain Reaction) assays. Moreover, we identified studies that detected antifungal resistance genes (e.g., FKS for echinocandins and ERG11 for azoles). The analyzed outbreaks were from all continents, which confirms the capability of this yeast to spread between humans and to contaminate the environment. It is important that real-time PCR assays were developed for accurate and affordable detection by all laboratories, including the detection of antifungal resistance genes. This will allow the fast and efficient implementation of stewardship programs in hospitals.

## 1. Introduction

In 2022, the World Health Organization (WHO) Antimicrobial Resistance Division released a report in which the rising invasive fungal diseases were compared to drug-resistant bacterial infections. Both bacteria and fungi represent important medical issues, especially fungal infections, which are challenged by limited access to quality diagnostics and treatment.

The pathogens included were classified and then categorized into three priority groups (critical, high, and medium). The critical group includes *Cryptococcus neoformans*, *Candida auris*, *Aspergillus fumigatus*, and *Candida albicans*. The WHO endorses including fungal diseases and priority pathogens in medical (clinical) and public health training programs and encourages optimizing the mycology diagnostic capacity to manage fungal infections and to perform surveillance [1]. Although taxonomical changes may prove difficult to adapt to, the recent fungal reclassification is another step in underlining the importance of fungal resistance and the possible emergence of novel pathogens. Recently, a change in the nomenclature of fungi has been underway, reclassifying fungal species into new genera based on antifungal resistance and thermotolerance [2]. The WHO priority fungi list mentions two such reclassified species: *Nakaseomyces glabrata* (*Candida glabrata*) and *Pichia kudriavzevii* (*Candida krusei*) in the high and medium priority groups, respectively [1].

*Candida auris*, a critical priority fungal species according to the WHO, emerged as a yeast that is multidrug-resistant in the past decade. This species inhabits the surface of the human skin and hospital settings, leading to the occurrence of healthcare-associated epidemics and invasive candidiasis. The initial genetic studies identified four primary clades, which were designated after their corresponding geographical locations: South Asia, East Asia, Africa, and South America. These clades are referred to as clade I, clade II, clade III, and clade IV, respectively, and are differentiated by at least ten thousand single-nucleotide polymorphisms (SNPs) between any two clades. In 2022, a fifth clade, from Iran, was confirmed by whole-genome sequencing [3].

The first case of *Candida auris* was isolated in 2009 from a sample of ear discharge from the external ear canal of a 70-year-old Japanese woman, as reported by Satoh K et al. [4]. The authors proposed this new species based on a single isolate, after the comprehensive description of the fungus (Latin diagnosis and description of *Candida auris*). The phylogenetic tree of JCM15448T in the 26S D1/D2 domain (AB375773) and ITS region (AB375772) sequences were also presented in that paper [4].

The first seven cases of *C. auris* identified in the USA were presented by the CDC (Centers for Disease Control and Prevention) in 2016, by Vallabhaneni S et al., which included the analysis of five blood samples, one urine sample, and one ear pus collection [5]. The authors mentioned the importance of using matrix-assisted laser desorption/ionization time-of-flight mass spectrometry (MALDI TOF) or sequencing the D1-D2 region of the 28S ribosomal DNA, as biochemical assays, for example, VITEK 2, seem not to be sensitive enough for the accurate detection of this fungal species. By whole-genome sequencing (WGS), the authors detected high similarities between those seven strains, and then concluded that *C. auris* could be considered an emerging pathogenic fungus, multidrug-resistant, with some strains having elevated minimum inhibitory concentrations (MICs) to drugs belonging to all major classes of antifungal medications. The authors also reported that this fungus has the capability of spreading within healthcare settings [5].

Two years later, Chow NA et al. published in ‘The Lancet’ the results of a WGS—molecular epidemiological survey of *C. auris* in ten US states. The authors found that all *C. auris* isolates isolated from the patients assessed in the USA belonged to one of the four known clades (African, South American, East Asia, and South Asia), suggesting several introductions of *C. auris* into the USA. Molecular epidemiology has provided key insights into the origin and transmission dynamics of *C. auris* in US healthcare facilities. The authors underlined that measures are needed in preparing for outbreaks and identifying this new multidrug-resistant yeast in order to contain its spread and transmission within the USA [6].

Another very recent ‘Lancet’ paper from 2023 published the characteristics of the five cases of coinfection from a southern Nevada dual-clade outbreak of *Candida auris*. The coinfections were isolated from different samples (two from the axilla, blood, and urine; two from the axilla and urine; and one from urine and wound sample). For the strains isolated from blood and urine samples, the capsofungin MIC value was determined. The available phenotypic susceptibility data showed that, in the isolates with a mutation in the FKS1 gene, the MIC values for caspofungin were elevated when compared to wild-type isolates [7].

The aim of this review was to summarize the recent findings regarding *Candida auris*’s molecular diagnosis in worldwide outbreaks registered in the last five years. To date, there is no FDA-approved assay for this fungus, and therefore, hospital microbiology laboratories should be properly informed, from a scientific and logistic point of view, regarding which assay is most suitable to be implemented for the fast and accurate diagnosis of *C. auris*.

## 2. Worldwide Guidelines Elaborated to Control *C. auris* Outbreaks

Many countries have elaborated new protocols for the early detection, treatment, and prevention of *C. auris* infections. Boyce JM. et al., from the USA, stated in a recent paper that the potential infection risk of this newly described fungus is on the same level as SARS-CoV-2 and mpox. The authors highlighted the importance of utilizing efficient alcohol-based hand sanitizers, as there is still research into the factors impacting hand hygiene practices [8]. A collaborative study that included almost all the countries from Europe published the initiative of The European Confederation of Medical Mycology (ECMM) with the intention to share their broad expertise on difficult-to-treat invasive fungal infections, and the advice will follow recent guidelines, including EQUAL (European QUALity (EQUAL) score). This Mycology Confederation wants to improve treatment outcomes and increase survival rates for difficult-to-treat invasive fungal infections [9]. One year prior to this study, the same team of Koehler P. et al. developed the international *Candida* Registry (FungiScope™ CandiReg) to allow contemporary multinational surveillance. The registry was thought to be necessary, given the previously reported outbreaks of *Candida auris* and the potential of this pathogen to produce difficult-to-treat infections. The European Confederation of Medical Mycology includes 28 countries, and with the release of this online registry, it intends to promote international collaboration for an optimal management of *Candida* infections [10]. In a comparative study for the period of 2013–2017 in a hospital from Johannesburg, South Africa, the authors highlighted the emergence of *Candida auris*, whose incidence increased from zero in 2013 to 11% in 2017. The researchers suggested that empirical antimicrobial therapy should be guided by an intensive care unit-specific antibiogram, and empirical antifungal therapy with amphotericin B or micafungin is adequate for patients that are at a high risk of invasive *Candida* infections [11]. Ong CW et al. published in 2019 a review about the position of the Australasian Society for Infectious Diseases regarding the most suitable assay for *Candida auris* identification, MALDI TOF or sequencing, and stated that phenotypic methods could lead to misidentification. The Australasian researchers recommended echinocandins as first-line therapy for infection in adults and children ≥2 months of age. For neonates and infants <2 months of age, they recommended amphotericin B deoxycholate. Also, they suggested that composite swabs of the axilla and groin should be collected for screening patients for *C. auris* [12]. Another guideline was elaborated by researchers from South Africa, which encountered 100 South African hospitals with cases of infections caused by *Candida auris*. Govender NP et al. released 18 practical recommendations, including the statement that the reference identification method should be molecular identification and the standardized broth microdilution (BMD) method should be used as a reference method for antifungal susceptibility testing. This guideline offers information regarding antifungal stewardship (e.g., diagnosis, stewardship, duration of therapy for sepsis, and antifungal consumption) [13].

All the above guidelines, produced by researchers from all continents, support the need for the immediate notification of *C. auris* isolation to clinical and infection control teams to prevent internal and inter-center transmission and to ensure the proper surveillance and prevention of infection of patients who are already colonized and can develop an infection.

## 3. Molecular Assays Used for the Active Surveillance of *C. auris* Outbreaks

In order to select the papers that would be included in this narrative review, we analyzed the molecular assays performed by different authors to assess *Candida auris* outbreaks in the last 5 years (2019–2024). Our search was initiated on 12 February 2024, and we searched the most prominent scientific databases, Web of Science-Clarivate and PubMed. For this process, we used the following keywords “*Candida auris*; outbreak; molecular detection”. Thus, 68 results were revealed for the last 5 years. A total of 6 papers were excluded as their topic and scientific relevance did not fit the scope of our review, and thus, 62 papers were included in the present narrative review. 

From these 62 articles, only 25 represented original research papers [14,15,16,17,18,19,20,21,22,23,24,25,26,27,28,29,30,31,32,33,34,35,36,37,38]. 

In Table 1, we include 34 papers, and it can be noticed the synthetic approach on the molecular detection of *Candida auris* in recent worldwide outbreaks (Table 1). We can observe that outbreaks with these new fungi have been documented worldwide: Europe (Spain, The Netherlands, and UK), Asia (Singapore, Japan, Iran, Turkey, China, Kuwait, and Lebanon), Africa (South Africa), USA, South America (Brazil), and Central America (Honduras). The most numerous studies for the analyzed period were from the USA (5/23), followed by Spain (3/23). The assays used were very diverse, from PCR with pan-fungal primers to MALDI-TOF MS, WGS of the microorganism, commercially available Eazyplex^®^ *Candida auris* kit, AmplexDiagnostics GmbH, Buseck, Germany, and a genosensor for *C. auris*. In total, these 23 analyzed papers tested 2770 clinical yeast samples for *C. auris*. One of the authors’ study directions was to detect the new fungus directly from pathological samples, before previous DNA extraction, to improve the speed of the detection process. Another direction was to develop fully optimized assays, with high sensitivity and specificity, suitable for being commercially available. A very interesting and clinically useful approach was to detect, by real-time PCR or WGS, the genes that are known to be responsible for antifungal resistance: Y132F in ERG11, as the most widespread mutation associated with azole resistance, and S639P in FKS1 for echinocandin resistance. For antifungal susceptibility testing, the authors used different assays, such as the Vitek^®^ 2 System (Biomerieux, Denver, CO, USA) or Micronaut (Bruker, Billerica, MA, USA). The heterogeneity of the molecular assays used to detect *C. auris* can easily be observed, with different characteristics that are listed in Table 1. Of course, it would be ideal for healthcare providers to use clinically validated tests, recommended by official organizations in the field.

In our analysis, we identified 25 papers, of which 6 used WGS + MALDI-TOF, 3 used MALDI-TOF + PCR/CULTURE, 11 used PCR, and 5 used other methods for *C. auris* detection.

The most comprehensive assay is WGS, of course, being a method that offers detailed genetic information, but has the disadvantage that it requires special and expensive laboratory equipment to be performed. MALDI-TOF is a very fast and accurate identification assay, and it needs to be followed by antifungal susceptibility testing. Real-time PCR allows for fast detection, and it was the most frequently used assay, probably because many laboratories, after the emergence of the SARS-CoV-2 pandemic, acquired the necessary equipment. More than simple detection, real-time PCR, which is able to detect antifungal resistance genes, seems to be even more suitable for fast diagnosis during *Candida auris* outbreaks.

## 4. *Candida auris*: Multidisciplinary Involvement

In the outbreak cases that we analyzed in this paper, there were diverse samples originating from ear aspirates, skin, blood samples, mucous membranes, and surfaces samples. We wanted to understand to which extent *C. auris* can be involved in other types of infections. *C. auris* was identified in a low-birthweight (800 g), preterm neonate in Italy in 2021. The authors recommended antifungal prophylaxis in low-birth-weight, preterm neonates with micafungin to avoid an unfavorable evolution [39]. In a review by Sokou R et al. from 2024, 24 studies in neonates that were positive for this yeast were identified. The authors underlined the need of understanding *Candida auris*, whose pathogenesis is very important for developing effective strategies to control and prevent neonatal infections caused by this pathogen [40].

Interestingly, related to *C. auris* pathogenesis is the paper of Vila T et al. in the field of dental medicine. The authors used murine models of infection to comparatively evaluate the host niche-specific pathogenic potential of *C. auris*, and they found that *C. auris* adheres more avidly, forming robust biofilms on catheters implanted in mice [41]. In the same context of analyzing pathogenesis, Wang TW et al. used a comprehensive transcriptional analysis to identify key cell surface adhesins. They used a mouse model of catheter infections and they identified that adhesins were highly upregulated in the aggregative phenotype during in vitro and in vivo grown biofilms. This could explain the flexibility of *C. auris* and its rapid adaptation to the environment, potentially impacting persistence and virulence [42]. Magnasco L et al. was interested in the frequency of *C. auris* outside a highly endemic setting and, after analyzing all the cases from their 1000-bed hospital in Genoa, Italy, they recommended screening at discharge from the endemic ward(s), even in the case of a recent negative result [43]. Prażyńska M et al. reported a case of meningitis positive for *C. auris*, identified by MALDI-TOF and WGS [44]. Theodoropoulos NM et al. reported a cluster of six patients, two being recipients of liver transplants, positive for *C. auris* [45]. Solid-organ transplant recipients are at high risk of developing *C. auris* bloodstream infections, as the authors from Florida, USA, found *C. auris* in five solid-organ transplant recipients (one heart, three liver, and one combined liver–kidney transplants) [46].

Regarding *C. auris*’s involvement in dental pathology, research in this field is still in its beginning. A September 2023 study by Alfaifi et al. presented a retrospective analysis, over a period of 2 years, from the institutional experience of a single center. In this study, 310 samples of saliva from patients experiencing symptoms or who had evidence of oral candidiasis were tested for the presence of yeasts. The result was that *C. auris* was not isolated from any of these samples, leading to the conclusion that it is not a common colonizer of the oral cavity. Also, the authors identified eight other studies of *Candida auris* being detected in more than 30 ocular, nasal, pharynx, and tracheal samples [47]. Another research team reviewed papers about the composition microbial biofilm found on dentures. While there is an important body of data on the microbial composition of dental plaque, the data published about denture plaque are limited. These artificial surfaces, depending on the materials in their structure, can be more prone to colonization by some microorganisms, including *Candida* species, leading to denture-associated stomatitis. *C. albicans* is the most well-studied organism found in denture plaque, followed to a lesser extent by other yeast species, and regarding *C. auris*, the authors note that, to date, no study has reported it to be found in denture microbial biofilms [48]. Transient oral colonization with this multidrug resistant fungus, in non-human mammals, has already been reported, and the first such case of *C. auris* oral colonization in a pet was published in 2024. The health implication of this oral colonization in pets is that zoonotic transmission to humans may be possible, given the long period of time that this pathogen can remain on surfaces [49]. In the case that human transient oral colonization is also reported, screening for oral *C. auris* may be recommended in the future before dental procedures, due to the possibility of hematogenous spread and even severe systemic infections in immunosuppressed patients.

This short summary showed that *C. auris* is capable of infecting patients from different medical wards (surgical, infectious disease, neonates, solid organ transplants, and dentistry).

## 5. *Candida auris*: Antifungal Resistance Mechanisms

The antifungal resistance phenomenon is well-known for *Candida auris*. We analyzed which were the most frequently identified resistance mechanisms in original studies published in PubMed in the last year (2023–2024). We identified 10 original papers, 4 of them published by researchers from the USA. The antifungal resistance mechanisms identified were diverse: resistance to phagocytosis by macrophages, mutations that explain increased fluconazole and voriconazole resistance (*ERG11* and *TAC1B* genes), missense substitutions in *FKS1* for echinocandin resistance, and the possibility of using the proton pump inhibitor lansoprazole to restore the activity of amphotericin B (AmB) in resistant isolates. Identifying with high precision the antifungal resistance mechanisms could lead to opportunities in the development of novel antifungal drugs against *C. auris* and could help in tracking the spread of *Candida auris* between cities and countries by highlighting the genetic relation between strains (Table 2) [14,50,51,52,53,54,55,56,57,58].

## 6. *Candida auris* Antifungal Stewardship

Kriegl L et al., in a paper published in March 2024, mentioned *C. auris* as one of the most important WHO fungal priority pathogens. The authors presented an antifungal therapy that was shown to have a good activity against these fungi. Echinocandin therapy is currently considered the first choice when treating an invasive *C. auris* infection, with the novel echinocandin, rezafungin, having very good in vitro activity against C. auris. For the *C. auris* isolates with FKS mutations encoding for echinocandin resistance, ibrexafungerp treatment seems to be effective. Another promising therapy is the one with fosmanogepix, as it proved its efficiency in MDR (multidrug-resistant) *C. auris* candidemia and in mice and rabbit models of cerebral spinal fluid infection therapy [59].

Several papers reported on the relationship between the *C. auris* clade and antifungal resistance, which were included in a recent review by Sharma and Kadosh, who found that isolates belonging to clades I, III, IV, and V show resistance to fluconazole and cross-resistance to echinocandins and amphotericin B, with even the possibility of pan-drug-resistant strains. Clade II, found predominantly in Korea and Japan, was found to have a lower degree of antifungal resistance [60].

The WHO guideline mentions surveillance actions, interventions, and strategies, in which there is a crucial necessity of antifungal stewardship activities by limiting the inappropriate use of antifungals and antibiotics, avoiding antifungal’s overuse and selecting the best drug, dose, and duration, and appropriate cases where they are needed. Also, it is mandatory to develop standard procedures to optimize the diagnosis of fungal infections that also include pathogens with outbreak potential and increase the capacity for outbreak detection, reporting, and response [1].

## 7. Conclusions

*Candida auris* was involved in more than twenty outbreaks, happening in all continents, in previous years. Given the fact that there is no specific prevention mechanism available, the fast detection of this pathogen in patient samples, without prior DNA extraction, can prove to be a salutary step in the management of these cases. In this way, by ensuring fast detection, safety procedures for limiting the spread of *C. auris* in the hospital environment could be implemented. From all the identified assays (e.g., WGS, MALDI-TOF, and PCR) to diagnose *C. auris* outbreaks, the most appropriate for routine diagnosis is, from our point of view, real-time PCR, due to its detection speed, the possibility of also identifying resistance genes, and readily available laboratory equipment. Commercially available kits should be validated by each laboratory before implementing them in routine diagnosis. Moreover, studies that already identified the resistance mechanisms to antifungals used for the treatment of these infections will favor stewardship strategies, contributing to preventing future resistance emergence.

## Figures and Tables

**Table 1:** Molecular detection of *Candida auris* in recent worldwide outbreaks.

Author, Year, Country	Number/Type of Samples	Assay Used for *C. auris* Detection	Results	Clinical Interpretation/Importance
WGS + MALDI-TOF				
Erturk Sengel B et al., 2023, Turkey [14]	One clinical *C. auris* isolate from blood cultures	MALDI-TOF Whole-genome sequencing (WGS)	*C. auris* belonged to clade I (South Asia). Resistance-related genes: CDR1, TAC1b, and ERG11	Mutations are responsible for azole resistance. The isolate was resistant to amphotericin B and caspofungin, with no corresponding mutations.
Kekana D et al., 2023, South Africa [15]	287 isolates of cultures confirmed *C. auris* from the neonatal unit	Whole-genome sequencing	181/188 isolates with fluconazole MIC > 32 µg/mL had ERG11 mutations.	The isolates from the neonatal outbreak belonged to clade III. Clade III isolates showed VF125AL substitutions and clade IV isolates had K177R/N335S/E343D substitutions.
Reslan L et al., 2022, Lebanon [16]	28 *C. auris* isolates from different samples (blood, urine, skin swabs, etc.)	MALDI-TOF identification Antifungal resistance using the Vitek-2 System and E-test WGS to determine clade distribution	The isolates were resistant to fluconazole and amphotericin B, but fully susceptible to echinocandins. *C. auris* genome belonged to the South Asian clade I.	All isolates belonged to clade I. This method allows for an appropriate and rapid diagnosis to contain spread and outbreaks.
Chow NA et al., 2020, USA [17]	304 *C. auris* isolates from 19 countries on six continents	Whole-genome sequencing	Mutations: Y132F in ERG11 S639P in FKS1 High rates of antifungal resistance in clade I.	Global collaboration: genome evolution of isolates of *C. auris* from 19 countries. Estimated timing of the expansion of each *C. auris* clade and of fluconazole resistance.
Zhu Y et al., 2020, USA [18]	Samples from a *C. auris* outbreak in New York (NY) - 540 clinical isolates, - 11,035 patient surveillance specimens, - and 3672 environmental surveillance samples	MALDI-TOF MS Real-time PCR Culture on selective/nonselective media for the recovery of *C. auris* Susceptibility testing Sanger sequencing of the internal transcribed spacer (ITS) and D1/D2 regions of the ribosomal gene for *C. auris* genotyping	The study identified and confirmed *C. auris* in: - A total of 413 clinical isolates; - A total of 931 patient surveillance isolates. Identification of: - A total of 277 clinical cases; - A total of 350 colonized cases–proof of higher colonization rates in nares than in axilla/groin. Predominance of the South Asian clade I with intrinsic resistance to fluconazole, voriconazole, amphotericin B, flucytosine, and echinocandins.	Greater regional prevalence and incidence of *C. auris*. Strategies to deploy better detection tools in outbreaks.
Sekizuka T et al., 2019, Japan [19]	7 *C. auris* strains from patients with otitis media	Whole-genome sequencing	All isolates belonged to clade II. Each *C. auris* clade showed single nucleotide variations, clade-specific accessory genes, and copy number variations.	Gene duplication events in *C. auris* might contribute to antifungal drug resistance. Genomic structural variations in *C. auris* could be correlated to geographical dissemination, epidemiology, lesions produced, and antifungal resistance.
MALDI TOF + PCR/CULTURE				
Hernández Felices FJ et al., 2023, Spain [20]	51 pharyngeal and axillary–rectal swab samples	Eazyplex^®^ *Candida auris* kit (AmplexDiagnostics GmbH, Gars Bahnhof, Germany) for the rapid identification of *C. auris* Culture MALDI-TOF identification	The sensitivity, specificity, and positive and negative predictive values between the 2 assays were: 91.8%, 98.8%, 98.2%, and 94.5%, respectively.	Eazyplex^®^ *Candida auris* showed acceptable results: - Single test; - Short time; - High specificity and positive and negative predictive values.
Tan YE et al., 2019, Singapore [21]	7 *C. auris* isolates	MALDI-TOF identification ITS sequencing VITEK^®^2 System antifungal susceptibility testing	Three clades were identified: South Asia (71.4%), South America (14.3%), and East Asia (14.3%). Three isolates (42.9%) were multidrug-resistant.	Introduction of *C. auris* into Singapore was possibly over multiple episodes and from different sources. The VITEK^®^2 System version 8.01 software analyzed in the study had limited ability in identifying *C. auris*.
Taori SK et al., 2019, UK [22]	Samples of 34 patients infected or colonized with *C. auris*	MALDI-TOF identification PCR amplification with further sequencing VITEK^®^2 System antifungal susceptibility testing	*C. auris* bloodstream infections showed no significant difference in the survival probabilities compared to other candidemias.	Outbreak control cost in excess of GBP 1 million and GBP 58,000/month in the subsequent year. *C. auris* outbreaks can be controlled by robust infection control strategies but can be expensive.
PCR				
Naeimi B et al., 2024, Iran [23]	221 ear aspirates from otomycosis patients	Conventional PCR Pan-fungal primers for screening the samples Samples positive for *C. auris* were validated by sequencing	5 samples positive for *C. auris* (5/189; 2.6%). 4/5 cases reconfirmed by sequencing.	PCR assay can be successfully applied for the rapid and accurate detection of *C. auris* directly from patient samples.
Ramírez JD et al., 2023, USA [24]	30 axilla/groin samples positive for *C. auris*	DiaSorin Molecular *C. auris* Detection Kit	The analytical sensitivity was determined using standard calibration curves with cell-forming units of yeast (CFUs).	DiaSorin Molecular *C. auris* Detection Kit: - Has the potential to aid in controlling the outbreaks; - Allows the screening of drug-resistant *C. auris*.
Zhu Y et al., 2023, USA [25]	31 clinically sourced cases	TaqMan chemistry probe-based fluorescence melt curve analysis (FMCA) following asymmetric polymerase chain reaction (PCR)	F635C, F635Y, F635del, F635S, S639F or S639Y, S639P, and D642H/R645T mutations.	TaqMan chemistry probe-based FMCA allowd the rapid and accurate detection of FKS1 mutations.
Safari F et al., 2022, Iran [26]	439 yeast isolates and 590 clinical specimens	Conventional PCR Real-time PCR	4/590 clinical specimens (0.68%) were detected as positive by conventional PCR and 6/100 positive by real-time PCR, with 0/439 cases positive for *C. auris*	In Iran, *C. auris* is not a common etiological agent of superficial or systemic fungal infections.
Asadzadeh M et al., 2022, Kuwait [27]	49 *C. auris* isolates from immunocompromised patients	E-test Broth microdilution-based MICRONAUT-AM assay Mutations in hotspot-1 and hotspot-2 regions of FKS1 were detected by PCR amplification and sequencing	Fluconazole and amphotericin B resistance was detected in 44 and 4 isolates, respectively. Twelve *C. auris* isolates showed reduced susceptibility to echinocandins.	A novel mutation in FKS1 was identified. The clinical outcome for many *C auris*-infected patients was unfavorable.
Freitas BL et al., 2022, USA [28]	33 surveillance samples	Quantitative real-time PCR targeting the internal transcribed spacer 2 (ITS2) ribosomal gene	The assay was highly sensitive, with a detection limit of 10 CFUs per RT-qPCR.	*Candida auris* RT-qPCR assay could be a useful test in surveillance efforts to manage the transmission of live *C. auris* in healthcare settings.
Mulet Bayona JV et al., 2021, Spain [29]	113 swabs (pharyngeal or axillary–rectal) used for assay validation 136 pairs of pharyngeal and axillary–rectal swabs	AurisID^®^ kit, OLM Diagnostics, Braintree, United Kingdom for the direct detection of *C. auris* from surveillance samples without prior DNA extraction	The PCR method showed high sensitivity, specificity, predictive positive value, and predictive negative value (96.6%, 100%, 100%, and 98.2%, respectively).	AurisID^®^ kit allows a reduction in the diagnostic time for surveillance of *C. auris*. This quick detection method could be used to control *C. auris* outbreaks.
Ahmad S et al., 2020, Kuwait [30]	314 samples of *Candida auris*	PCR amplification and/or PCR sequencing of ribosomal DNA VITEK^®^2 System E-test PCR amplification and DNA sequencing of ERG11 and FKS1 genes	The proportion of *C. auris* in the bloodstream among all yeast isolates was higher (13.7%) in 2018 compared to 2014–2017 (1.7%).	*C. auris* spread in major hospitals across Kuwait. Y132F or K143R mutation in ERG11.
Jainlabdin MH et al., 2019, UK [31]	Isolates belonging to 8 different *Candida* species	Single-tube, dual channel pentaplex molecular diagnostic assay based on Multiplex Probe Amplification technology Post-amplification melting curve analysis allowed the identification of the infectious agent	The high multiplexing capacity, wide detection range, high specificity, and sensitivity of this MPA Candida assay indicate its potential for clinical diagnosis and for controlling and managing hospital outbreaks.	*C. auris* detection, as a well-characterized multidrug resistant outbreak strain, will facilitate rational therapy and antifungal stewardship.
Franco LC et al., 2024, USA [32]	282 screening samples from axial, inguinal, and/or other external body sites	A real-time qualitative PCR assay, developed in the laboratory, using the DiaSorin (Stillwater, MN, USA) *C. auris* primer set on the LIAISON MDX thermocycler with the Simplexa Universal Disc	The assay was highly sensitive and specific, with a limit of detection of 1–2 CFU/reaction. Performance of the *C. auris* real-time PCR assay compared to a culture as gold standard testing: Sensitivity: 100.0%. Specificity: 100.0%. Positive predictive value: 100.0%. Negative predictive value: 100.0%.	The study evaluated the performance of a commercial primer set and reagents utilized on a commercially accessible platform, which could be used as a high-throughput testing option for *C. auris* colonization screening.
Cerqueira FM et al., 2024, USA [33]	148 axilla and groin samples collected from high-risk patients, as well as environmental samples from the patient rooms	A laboratory-developed CAURIS-PCR assay implemented on the Hologic Fusion Open Access platform, validated in comparison to cultures and MALDI-TOF identification	100% sensitivity. 93.53% specificity. 50% positive predictive value. 100% negative predictive value.	The study shows that the CAURIS-PCR assay allows for rapid results, which may ultimately improve patient care during outbreaks of *C. auris* infections.
OTHER METHODS				
Zhang XR et al., 2023, China [34]	Experimental strains of *C. auris*	Recombinase-aided amplification combined with lateral flow strips (RAA-LFS)	*Candida auris* was accurately identified and differentiated from related species at 37 °C, within 15 min.	RAA-LFS has the advantage of the rapid clinical detection of *C. auris* and saving treatment time for the infected patients.
Ortiz B, et al., 2022, Honduras [35]	328 yeast isolates from clinical samples	Molecular approach: size polymorphisms of the hpw1 gene to identify the yeast species	Eleven species of *Candida* were identified in hospital wards, while *C. auris* was not found in any of the samples.	The method of *C. auris* detection is simple and could help in low-resource settings.
Guedes PHG et al., 2021, Brazil [36]	Urine samples containing different concentrations of *C. auris*	Gold electrode sensitized with specific DNA capture probe and ninhydrin as a novel DNA hybridization indicator The genosensor detected *C. auris* by differential pulse voltammetry and electrochemical impedance spectroscopy	The biosensor’s range of detection was evaluated to be 4.5 pg μL^−1^ of *C. auris*’s genomic DNA in urine.	The proposed system is a promising electrochemical device that allows for a more accurate detection of *C. auris*’s genomic DNA in biological samples.
Pla L et al., 2021, Spain [37]	14 samples of serum inoculated with *C. auris* isolates from 8 countries	A biosensor based on oligonucleotide-gated nanomaterials was used Nanopores in an anodic alumina scaffold filled with rhodamine B and different oligonucleotides are capable of specifically recognizing *C. auris* genomic DNA	*C. auris* could be detected at a concentration as low as 6 CFU/mL.	This test can provide a diagnostic result in clinical samples in one hour, without prior DNA extraction or amplification steps.
de Groot T et al., 2020, The Netherlands [38]	444 *C. auris* isolates investigated to identify genotypic diversity	Novel simple STR genotyping technique based on short tandem repeats in the *C. auris* genome Twenty-three STRs were selected and twelve were used to develop a STR typing assay.	The study identified the five major different *C. auris* clusters of South American, South Asian, African, East Asian, and Iranian origin and 40 distinct genotypes.	The test is a novel and simple STR genotyping technique. The performance of this STR-based genotyping technique was comparable to that of WGS.

**Table 2:** Identified antifungal resistance mechanisms for *Candida auris*.

Author, Year, Country	Assay	Resistance Mechanism Identified	Medical Importance
Salama EA et al., 2024, USA [50]	A panel of 727 FDA-approved drugs were screened to find a potential enhancer by minimum inhibitory concentration.	Proton pump inhibitor lansoprazole can restore AmB’s activity against *C. auris*.	This study should be followed by a comprehensive evaluation of lansoprazole as a cytochrome bc1 inhibitor for *C. auris*-resistant strains.
Yang B, et al., 2024, USA [51]	Two C. auris isolates underwent genomic comparison of their carbon metabolism pathways.	- There is a relationship between metabolism and drug tolerance. - B11221 strain from clade III resists phagocytosis by macrophages and exhibits decreased β-1,3-glucan exposure. - B11220, a susceptible strain from clade II, showed deletions of genes related to sugar assimilation.	Alternative sugar utilization and membrane composition positively correlate to drug tolerance and immune evasion, respectively.
Chen XF et al., 2024, China [52]	WGS to identify drug resistance mutations of 16 *C. auris* isolates.	Notable mutations: - Y132F in *ERG11* and A585S in *TAC1b*, which are possibly linked to increased fluconazole resistance. - F214L in *TAC1b*, related with a consistent voriconazole resistance.	This study highlights the potential transmission of *C. auris*-resistant isolates and underlines the need to explore variations related to antifungal resistance.
Misas E et al., 2024, Columbia [53]	WGS to identify drug-resistance mutations of 99 *C. auris* isolates.	Mutations in the *ERG11* and *TAC1b* genes are related to fluconazole resistance.	*C. auris* cases from different geographic locations exhibited high genetic similarity.
Hansanant N et al., 2024, USA [54]	New structural variants of occidiofungin were tested against *C. auris* strains.	Some chemical analogues showed higher activity compared to the natural ASP7 variant, against a panel of Candida species, including *Candida auris*.	This study shows the potential of developing new occidiofungin derivatives to combat resistant *C. auris* infections.
Li J et al., 2024, Switzerland [55]	RT-PCR analyses of UPC2 expression.	Upc2 regulates *ERG11* expression and activates the Mrr1/Mdr1 pathway.	Upc2 is plays a crucial role in the azole resistance of *C. auris*, via the regulation of the ergosterol biosynthesis pathway and activation of the Mrr1/Mdr1 pathway.
Wang Y et al., 2024, Canada [56]	An analysis of SNPs from 19 genes related to antifungal resistance and MIC values for 387 *C. auris* strains.	The study identified: - Four SNPs in genes encoding lanosterol 14-α demethylase and the catalytic subunit of 1,3-beta-D-glucan synthase *(ERG11* and *FKS1*) were common between clades, while others are clade-specific. - The experimentally confirmed Ser639Phe/Pro missense substitutions in *FKS1* are related to echinocandin resistance.	A further evaluation of the role of SNP in the mechanisms of drug resistance in *C. auris* is required, but this study draws attention to the high diversity of these mechanisms, which may pose important medical challenges.
Schikora-Tamarit MÀ et al., 2024, Spain [57]	An analysis of genomic variants of approximately 2000 isolates from six *Candida* species, including *C. auris*.	The study found *TAC1b*, *ERG11*, and *MRR1* in *C. auris* (related to azole resistance).	*C. auris* diversification likely took place before human colonization. Several candidate genes of resistance were found in the studied genomic variants.
Erturk Sengel B et al., 2023, Turkey [14]	WGS of a clinical isolate of *Candida auris* used to predict antifungal resistance and then compare to antifungal susceptibility results.	MaCa01 was found to be part of clade I and would most likely be highly resistant to fluconazole due to mutations in *CDR1*, *TAC1b*, and *ERG11*, and predicted to be susceptible or with low resistance to amphotericin B (AmpB) and echinocandins.	Although no mutations in *ERG2, ERG6,* and *FKS* genes were detected, the isolate exhibited resistance to AmpB and caspofungin according to the CDC’s provisional breakpoints. This resistance may be attributed to unexplained mutations.
Lohse MB et al., 2023, USA [58]	1990 FDA-approved or investigational drugs were screened to be repurposed as antifungals.	Extended exposure to clioquinol leads to a 2- to 5-fold increase in resistance, likely due to mutations in: - Transcriptional regulator *CAP1* (causing upregulation of the drug transporter MDR1). - The drug transporter *CDR1*.	*CDR1* mutation increased susceptibility to posaconazole; thus, a combination treatment with this drug and 8-hydroxyquinoline might prevent *C. auris* from developing resistance.

## Data Availability

Not applicable.
